# Walking the talk: evaluating the alignment between Australian governments’ stated principles for working in Aboriginal and Torres Strait Islander health contexts and health evaluation practice

**DOI:** 10.1186/s12889-020-09983-w

**Published:** 2020-12-03

**Authors:** Joanne N. Luke, Angeline S. Ferdinand, Yin Paradies, Daniel Chamravi, Margaret Kelaher

**Affiliations:** 1 Evaluation and Implementation Science Unit, Centre for Health Policy, Melbourne School of Population & Global Health, The University of Melbourne, 4/207 Bouverie Street, South Carlton, VIC 3053 Australia; 2 School of Humanities and Social Sciences, Deakin University, Burwood, VIC 3125 Australia

**Keywords:** Aboriginal, Torres Strait Islander, Indigenous, Evaluation, Policy, Health, Health planning

## Abstract

**Background:**

Stated principles in government policy documents serve as a set of values outlining how governments intend to work. As such, health planning principles should be reflected in health policy across the cycle of planning, implementation and evaluation. Such principles should be reflected in the process of governments commissioning and funding evaluation, and in the work of those commissioned to do evaluation on behalf of governments.

**Methods:**

We reviewed health planning policy documents to identify principles Australian State and Territory and National governments stated as being important to the work they do within Aboriginal and Torres Strait Islander health contexts. Evaluation tenders and reports relating to Aboriginal and Torres Strait Islander health policy, programs and service for the period 1-Jan-2007 to 1-Jan-2017 were retrieved and assessed as to whether they embedded principles governments state as important.

**Results:**

In Aboriginal and Torres Strait Islander health planning policy contexts, Australian governments outline *shared responsibility, cultural competence, engagement, partnership, capacity building, equity, a holistic concept of health, accountability,* and *evidence-based* as fundamental principles that will underpin the work they will do.

In total, we identified 390 publicly advertised evaluation tenders, but were only able to retrieve 18 tenders and 97 reports. Despite strong rhetoric placing importance on the abovementioned principles, these were not consistently embedded in tenders released by government commissioners, nor in reports largely commissioned by governments. Principles most widely incorporated in documents were those corresponding to Closing the Gap - *accountability, evidence-based* and *equity*. Principles of *holistic concept of health, capacity building, cultural competence* and *partnership* do not appear well applied in evaluation practice.

**Conclusion:**

Notwithstanding the tensions and criticism of current practice that sees dominant governments policing Aboriginal and Torres Strait Islander populations and defining what principles should inform health policy and evaluation practice, this paper reveals shortcomings in current evaluation practice. Firstly, this paper reveals a lack of transparency about current practice, with only 2% of tenders and 25% of reports in the public domain. Secondly, this paper reveals that governments do not ‘walk the talk’, particularly when it comes to principles relating to Aboriginal participation in health.

## Background

### Role of evaluation in Aboriginal and Torres Strait Islander health

The equal right of Aboriginal and Torres Strait Islander people to attain the highest standard of health and wellbeing is enshrined within the United Nations Declaration on the Rights of Indigenous People [1]. This declaration, to which Australia is a signatory, asserts that Indigenous peoples have the right to actively develop and determine health priorities and strategies and that governments have a responsibility to progressively assist Indigenous populations to realise full health, as well as take measures to implement programmes for monitoring, maintaining and restoring health [1]. These rights are relevant to all stages of the policy cycle from planning to implementation to evaluation.

In Australia, over recent decades there has been considerable policy investment by Commonwealth and State and Territory governments towards the goal of achieving health equity for Aboriginal and Torres Strait Islander people. This has coincided with a government discourse focused on addressing health disparities and building a robust evidence base of what constitutes effective policy, programs and services. However, despite such investment, there is concern that Aboriginal perspectives and, in particular, the Aboriginal community-controlled sector (ACCS) do not always drive or inform the determination of health and wellbeing policy priorities and strategies [2]. Despite peak Aboriginal bodies such as the National Aboriginal Community Controlled Health Organisation (NACCHO) and their State and Territory affiliates being strong community voices and advocates for the health and wellbeing of Aboriginal and Torres Strait Islander communities, the inclusion of these important perspectives across health planning, implementation and evaluation are not realised to their fullest potential [2]. For decades we have seen Aboriginal people and the ACCS call for greater collaboration, engagement and leadership across health systems to allow the ACCS to define the health and well-being benefits and outcomes for Aboriginal and Torres Strait Islander populations [3, 4]. These voices have asserted that Aboriginal and Torres Strait Islander ways of knowing, doing and being need to be central to the planning, delivery, implementation and evaluation of health policy, programs and services. Such perspectives continue to challenge the deficit framing of Aboriginal and Torres Strait Islander populations people and their health through their ongoing demonstration that Aboriginal people, communities and organisations have unique and valued knowledge, expertise and skills and these strengths are vital for realising optimal health and wellbeing [5–8].

Establishing a comprehensive and accessible evidence base with the full participation of the ACCS is at the core of effective evaluation practice in Aboriginal and Torres Strait Islander health. Effective evaluation practices not only increase evidence by providing positive examples of what works, but they also serve to identify and ameliorate or avoid unintended negative consequences of policies, programs and practices. Evaluation also increases transparency and accountability by ensuring that programs and services are adequately resourced. Both these points are particularly salient in Aboriginal and Torres Strait Islander contexts, given government policies and programs (and lack thereof) have had and continue to have an immensely devastating effect on the Aboriginal and Torres Strait Islander communities. However, despite the potential benefits of evaluation and a high level of investment in independent evaluation, findings from many evaluations are never publicly released. Without publicly available evidence, Aboriginal people and the ACCS will continue to report the burden of being over-evaluated and policy makers will report a lack of evidence.

### Evaluation practice in Aboriginal and Torres Strait islander health

In 2012, a Productivity Commission round table attended by the ACCS, non-government, government and academic sector highlighted the importance of evaluation in improving health outcomes for Aboriginal and Torres Strait Islander people [2]. This roundtable identified that there are numerous fundamental system design issues with current evaluation practice. In particular, they emphasised the need for greater involvement of Aboriginal people and the ACCS in the development and evaluation of programs and policies, more information about existing programs such as their objectives and associated program logic, better integration and resourcing for evaluation plans in the design of programs, as well as the need for a cohesive evaluation framework for evaluation of policies and programs intended to improve Aboriginal and Torres Strait Islander health and well-being [2].

Since 2012, there has been an increasing national investment in evaluation with the Commonwealth government committing $40 million to strengthen reporting, monitoring and evaluation of programs targeted at improving Aboriginal and Torres Strait Islander health and wellbeing. This was followed in 2017 by the Productivity Commission appointing an Indigenous Policy Evaluation Commissioner and the whole-of-government Indigenous evaluation strategy proposal by the Productivity Commission in 2019. This period has also coincided with increased conceptual research [9] and the development of several evaluation tools for use in Aboriginal and Torres Strait Islander contexts, including the ‘Ngaa-bi-nya Aboriginal and Torres Strait Islander framework’ and Lowitja ‘Evaluation framework to improve Aboriginal and Torres Strait Islander health’ [10–13].

### Aligning health planning principles to evaluation

Bainbridge et al. (2015) emphasise the importance of principle-oriented practice to maximise benefit for Aboriginal and Torres Strait Islander people [3]. However, for evaluation, this is complicated. There are the National Health and Medical Research Council (NHMRC) guidelines *Ethical Conduct in Research with Aboriginal and Torres Strait Islander Peoples and Communities: Guidelines for Researchers and Stakeholders* (2018) which highlight the six values of reciprocity, respect, equity, cultural continuity, responsibility and spirit and integrity as principles important for research. Although the guidelines do cover evaluation, they only cover the relationship between the evaluator and communities and individuals involved in the evaluations [14]. While this is appropriate in investigator-driven research, commissioners and program implementers are also important agents in evaluation in terms of relationships with the community, consultation associated with program development, program implementation and the dissemination of information. It is only recently with the Lowitja Institute’s ‘Evaluation framework to improve Aboriginal and Torres Strait Islander health’ and BetterEvaluation’s ‘Good evaluation practice in Aboriginal and Torres Strait Islander settings’ protocol, that there are national-level documents that talk to the responsibilities and accountabilities of commissioners in evaluation practice [13, 15]. Such frameworks continue to form part of larger global endeavours that seek to build on classic bioethics approaches to promote social justice in research and evaluation. Such frameworks recognise that some populations are made vulnerable in evaluation owing to imbalances in the power dynamics between the evaluated and those driving evaluations. Such imbalances play out in both neo-colonial contexts that we describe for Indigenous populations as well as in evaluations involving high income countries working in low- and middle-income countries [16].

In terms of principles specific to evaluation, an overarching intent of the 2019 Indigenous Evaluation Strategy initiated by the Australian Government Productivity Commission has been to develop a principle-based evaluation framework for Australian government agencies. Success in enacting change through these principles will require identifying and addressing existing barriers to the implementation of principles for working with Aboriginal and Torres Strait Islander health. In addition to improving transparency and accountability in evaluation reporting.

Nationally and across State and Territory jurisdictions, health planning policy documents include a set of principles that serve as a set of values outlining how governments intend to work in Aboriginal and Torres Strait Islander health contexts. Health planning principles are acknowledged by governments as being important to the work they do. As such, health planning principles should be reflected in health policy across the cycle of planning, implementation and evaluation. In evaluation, such principles should be reflected in the work of governments commissioning and funding evaluation, as well as of those commissioned to do evaluation on behalf of governments. This paper is a meta-evaluation of the extent to which principles for working with Aboriginal and Torres Strait Islander people are included in evaluations of programs to improve their health and well-being.

## Methods

Ethics clearance was obtained from the University of Melbourne Human Research Ethics Committee (ID 1750086.1).

The project was overseen by a project reference group (PRG), whose members included representation from evaluation end-users including the ACCS, the Lowitja Institute, the Department of Health, the Department of the Prime Minister and Cabinet, and the Productivity Commission.

Here we conduct an evaluation of current evaluation practice across Aboriginal and Torres Strait Islander health and wellbeing policy, programs and services 2007–2017. We do so by using a 2-step approach. Firstly, by determining what principles governments state as being important to the way they work in Aboriginal and Torres Strait Islander contexts, and then secondly by assessing whether these principles are embedded in evaluation tendering and evaluation practice. We do so as a means of holding governments to account and measuring transparency of current practice. These 2-steps are described below.

### Review of government planning documents: identification of principles

A review of National, and State and Territory health planning documents was conducted in March 2017 to identify the principles that governments state as important for working in Aboriginal and Torres Strait Islander health contexts. An online search of Commonwealth and each State and Territory health department website was carried out to locate current Aboriginal and Torres Strait health planning policy. Thematic analysis was used to review these policy documents to identify the principles that governments state as important. Principles appearing across two or more documents were included for review. Content analysis was then used to explore how these principles have been articulated across each of the government planning documents. Documents were reviewed by two researchers (MK and JL) and where there was any variance, discussion was had to reach consensus. Once the principles were agreed upon, the wording used within reports was analysed to draw out how governments articulated these principles.

### Identification of government tenders and evaluation reports

Evaluation tenders are issued by commissioners of evaluations and detail the requirements of an evaluation and the obligations and responsibilities of the evaluator. Two sites were searched for tenders to evaluate programs in health and wellbeing: AusTenders.com and Tenders.net. The timeframe for the review was January 2007 to January 2017.

While AusTenders.com can be searched directly, Tenders.net does not list expired tenders. A special request was made for an offline search to be conducted and the results sent by email. In searching for relevant tenders, a broad definition was given to ‘health’ and ‘wellbeing’ in order to include evaluation in related fields, such as education, justice and sport. The search resulted in a large number of hits, with more than 12,000 hits returned from a search of the AusTender site. Search results were truncated to include only minimal information, so it is not possible to see the full material of the tender that is being searched. However, with the number of hits returned, it is possible that the words ‘Indigenous’ and/or ‘Aboriginal’ are included in a standard phrase in every Australian Government tender, which led to all tenders appearing as search results. The initial advice from Tenders.net was that a preliminary search showed 1864 matches; however, the final spreadsheet provided had 3441 results. While the representative from Tenders.net advised that the dataset included all public tenders listed on the AusTender site, as well as other sites, the AusTender search returned many results that did not appear in the Tenders.net spreadsheet. All search results from Tenders.net and AusTender were examined. After elimination of duplications and results that did not fit the criteria, 381 individual records were included. A further nine evaluations were identified from the website of the Australian Indigenous Health*InfoNet*, bringing the total records included to 390.

Despite the fact that all tenders are publicly listed initially, none of the tendering organisations nor the sites for publicly listing tenders keep a repository of tender information once it has been let. Tender documents were therefore located by contacting the person listed as being responsible for the tender. If the person was no longer available, the department responsible for the tender was contacted.

### Review of government tenders and evaluation reports

We reviewed tenders as a means of examining commissioning practice and reviewed evaluation reports as a means of examining evaluation practice.

Evaluation tenders and reports were reviewed to see if they included health planning principles that governments state as important. We then deductively identified how these were articulated by governments. For tenders, whole documents were reviewed with a focus on the selection criteria as these sections detailed how the evaluation should be conducted. For evaluation reports, whole documents were also reviewed with focus on the methodology, evaluation questions, outcome measures and program logics, as these provided information on how the evaluation was done and what was evaluated.

For reporting, we present the percent of tenders and reports underpinned by the principles identified in government planning documents.

## Results

### Review of government planning documents: identification of principles

In total, seven health policy planning documents were retrieved for the Commonwealth (national) government, and the States and Territory governments of Victoria (VIC), New South Wales (NSW), Queensland (QLD), Northern Territory (NT), South Australia (SA) and Western Australia (WA). At the time of the review neither Tasmania nor the Australian Capital Territory had a current health plan for Aboriginal and Torres Strait Islander populations. All health plans had a list of stated principles, except for the Northern Territory health plan, where the Strategic Directions have been used as a surrogate.

Table 1 outlines the stated principles that governments considered important in their health plans. Review of health planning documents revealed that shared responsibility, cultural competence, engagement, partnership, capacity building, equity, accountability, evidence-based, and a holistic concept of health were stated as principles of importance in two or more of the health planning documents. The principles of ‘recognition of diversity’ as well as ‘resourcing’ appeared in only one document, so were not considered in our analysis. The principle of partnership was in all seven health plans, while engagement and cultural competency were in all but one and evidence-based was specific to the VIC and the NT plans only. The rest of the principles were in around half of the plans.

**Table 1:** Stated principles underpinning National, State and Territory health policy planning

	National [17]	NSW [18]	NT [19]	QLD [20]	SA [21]	VIC [22]	WA [23]
Shared responsibility	Yes				Yes	Yes	Yes
Cultural competence		Yes	Yes	Yes	Yes	Yes	Yes
Engagement	Yes	Yes	Yes		Yes	Yes	Yes
Partnership	Yes	Yes	Yes	Yes	Yes	Yes	Yes
Capacity building			Yes	Yes	Yes	Yes	
Equity	Yes		Yes				Yes
Accountability	Yes				Yes	Yes	Yes
Evidence-based			Yes			Yes.	
Holistic concept of health	Yes		Yes		Yes		Yes

The articulation and interpretation of principles differed slightly across the health planning documents. For example, Table 2 describes how the principles have been articulated by governments. It is these broad understandings of the principles that we have evaluated for in practice. If an evaluation incorporated any of the definitions it was considered to embed that principle.

**Table 2:** Articulation of principles by governments across National, State and Territory health policy planning documents

*Shared responsibility*	The concept of shared responsibility featured in National, VIC and WA health planning documents. In WA it was articulated that health is ‘everybody’s business’ and in VIC the ‘responsibility for all’ in the health sector. Nationally, the concept of shared responsibility was extended to include Aboriginal and Torres Strait Islander people as well as governments and health services, through the concept of having a ‘shared ownership’ of health initiatives. Overall, the principle of shared responsibility emphasises that governments and health organisations need to be accountable, responsive and inclusive to Aboriginal and Torres Strait Islander needs.
*Cultural competence*	This principle featured in all State and Territory health plans. Cultural competence, first defined by Cross et al. (1989) and adopted by the NHMRC is defined as “a set of congruent behaviours, attitudes, and policies that come together in a system, agency, or among professionals that enable them to work effectively in cross-cultural situations” [24]. In the years since, there has been a focus on related concepts such as cultural awareness, cultural security and cultural safety, with critique by Aboriginal scholars highlighting a preference cultural safety with its requirements for broader systemic change [25, 26]. Health planning emphasised that factors desirable to the health systems included constructs such as ‘culturally secure’ (WA, NT), ‘cultural respect’ (SA, VIC, NSW, QLD), ‘culturally safe’ (NT), ‘culturally sensitive’ (QLD), ‘cultural recognition’ (NSW, QLD), ‘culturally responsive’ (QLD, VIC), and ‘culturally accessible’ (WA) Planning emphasised strengthening the capacity and capabilities of the health system to deliver culturally safe, secure and accessible health services, and of practitioners being respectful, sensitive, reflective and responsive to the views, traditions, values, expectations, worldviews and ways of working of the many diverse Aboriginal and Torres Strait Islander cultures that may be different from their own.
*Engagement*	Engagement was in six plans but not the QLD health plan. The articulation of engagement varied greatly across documents from ‘consultation’ and ‘input’ (VIC) to ‘participation’ and ‘involvement’ (National, NSW) to ‘participation to take back control’ and ‘responsibility’ (WA), through to full acknowledgement of ‘community control’ (SA). Despite inconsistent articulations of engagement, all six plans highlighted the importance of Aboriginal voices in health planning and delivery as well as the rights of Aboriginal people, communities and organisations to have control over decisions that impact on their health and wellbeing. In National and VIC planning, it was highlighted that it was governments who had a responsibility to expand opportunities for better engagement and collaboration.
*Partnerships*	The principle of partnership was embedded in all health planning documents, but there was not consistency regarding who needed to be a partner. Health plans referred to the importance of partnerships between Commonwealth and the State and Territory governments (National, WA), as well as between governments and Aboriginal people (National, QLD, NT), governments and communities (QLD, SA, NT, NSW) governments and Aboriginal organisations (National, VIC, NSW, WA) as well as with governments and other service providers and organisations (QLD, WA, SA, NT). Health planning detailed that partnerships involved governments and other stakeholders (Aboriginal and dominant organisations) actively establishing relationships and building effective long-term partnerships where there is collaborative ‘knowledge exchange’, ‘priority setting’, ‘information sharing’, ‘pooling of resources’ and ‘two-way skill transfer’. Partnerships were framed as important to ensuring Aboriginal voices, priorities and perspectives are reflected in policy and program design, planning, development, implementation and evaluation.
*Capacity building*	VIC, SA, QLD and NT health planning all had a capacity building principle, but there were not consistent articulations regarding whose or what capacity needed building. In VIC, the NT and SA capacity building operated from a deficit standpoint where emphasis was on building Aboriginal and Torres Strait Islander capacity, rather than recognising the existing community strengths and expertise. Here, capacity building entailed provision of skills, information or knowledge so that Aboriginal individual, families, communities, or organisations could be more responsive, manage change and/or maintain resilience. In QLD and the NT, the limitations of the health system were acknowledged, where capacity building drew upon strengthening the workforce and health system to provide more culturally responsive services.
*Equity*	Equity was a recognised human rights imperative and was understood as the offering of equal opportunities for health through the provision of available, accessible (physically and culturally), acceptable, quality, responsive and inclusive programs and services. The principle of equity was embedded within National, WA and NT documents and largely pertained to the reorientation of services so they were inclusive to the needs of Aboriginal and Torres Strait Islander people.
*Accountability*	Accountability was embedded in half of the health plans. However, there were not consistent articulations about who needed to be accountable and what for. Those required to be accountable to Aboriginal and Torres Strait Islander people included government (National, SA), the health sectors (VIC, WA), community organisations (SA) and mainstream health services (SA). Health plans stated that governments are accountable for monitoring and evaluating health activities, establishing measures of success, developing genuine and meaningful planning and service development partnerships, transparency in the allocation and use of public funds, and being responsive to performance. The health sector as a whole had accountability to lead and deliver health outcomes.
*Evidence-based*	Evidence-based was a guiding principle within the VIC and NT health plans. Evidence-based approaches were articulated as those that use evidence to inform health decision-making, policy and program design. Evidence-based approaches were presented as a way of ensuring that policy and programs are appropriate and effective, so they are positioned to deliver desired outcomes.
*Holistic concept of health*	Health plans for WA, SA, NSW, and VIC all had holistic concept of health as a stated principle. A holistic approach incorporates an understanding of the NACCHO definition of health a*s ‘not just the physical well-being of an individual but… the social, emotional and cultural well-being of the whole Community in which each individual is able to achieve their full potential as a human being thereby bringing about the total well-being of their Community. It is a whole of life view and includes the cyclical concept of life-death-life’* [27] The SA, NSW and WA articulations of holism also drew on social determinants approaches where there is recognition that health systems, racism, history of dispossession, and loss of land and heritage, food, water, housing, unemployment, contribute to health outcomes and need attention. Articulations of social determinants also drew on strengths of Aboriginal culture, spirituality, family and community and the importance of country and how these impact on health.

### Tender review: evaluation of current evaluation tendering practice

In total, 390 tender records were identified. Of these, 381 were retrieved through Tenders.net and AusTender and a further nine from Health*InfoNet* (healthinfonet.ecu.edu.au). Of the 390 identified records, we were only able to access 5% (*n* = 18) of relevant tender documents for review.

**Fig. 1:**
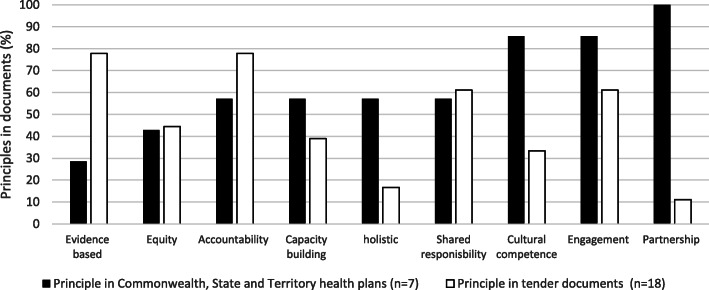
Stated principles in health planning relative to principles embedded in tenders

We found that principles stated as important in health planning by Australian governments (as described in Table 2) were not well or consistently embedded across the 18 evaluation tenders (Fig. 1). Principles featuring in health planning such as cultural competence, engagement and partnership did not always feature in tenders.

Despite partnerships between government and Aboriginal entities featuring in all Australian health plans, only 2 tenders (11%) indicated that a partnership approach to evaluation was required. Conversely, the principles of evidence-based and accountability were in most tender documents, despite these not being principles included in all health plans. Holism, cultural competence and capacity building were less frequent in tenders.

### Report review: evaluation of current evaluation practice

In total, 97 of 390 (24.8%) evaluation reports were retrieved using a web search. Of these, 83 were from government departments and 14 from other commissioning organisations that received government funding.

**Fig. 2:**
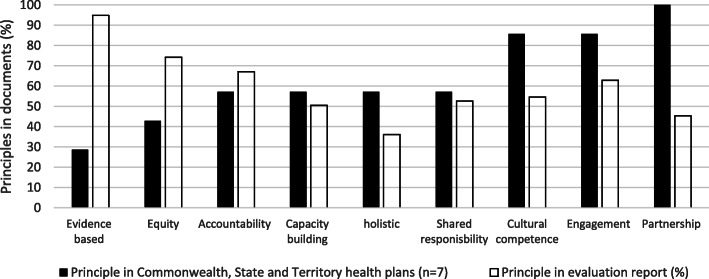
Stated principles in health planning relative to principles embedded in evaluation reporting

Consistent with tender findings, we found that principles stated as important in health planning were not well or consistently embedded across the 97 retrieved evaluation tenders (Fig. 2). We found an inverse relationship between the principles that government stated most frequently and those that were embedded in evaluation practice. For example, evidence-based was a principle in only 2 of 7 health plans, yet underpinned nearly all evaluations. Conversely, partnership was stated by all governments as a principle underpinning the work they do, yet was evident in only half of evaluations. We note that the 2007–2017 period coincided with the overarching ‘Closing the Gap’ policy led by the Coalition of Governments (National, State and local), to the large exclusion of the ACCS.

Across both evaluation tenders and reporting, the principle of holistic health was not well embedded, with most evaluations using biomedical understandings of health that focused on disease of individuals.

Table 3 revealed little difference in whether evaluation reports included identified principles across different governments (State/Territory and National), irrespective of the importance each placed on certain principles. Stating a principle as important did not mean that that jurisdiction better embedded it in practice. It is difficult to comment on jurisdictional differences owing to the small number of reports included, however these findings suggest the principle of partnership was less frequently embedded in National commissioned evaluations. Only 1 in 5 National reports had a partnership approach embedded.

**Table 3:** Percent of reports with principle embedded in evaluation reports

Jurisdiction (n of reports)	Evidence- Based %	Equity	Accountability	Capacity building	Holistic concept	Shared responsibility	Cultural competence	Engagement	Partnership
National (41)	–	83	68	–	34	63	–	63	19
NSW (11)	–	–	–	–	–	–	64	64	36
NT (17)	88	59	–	47	29	–	41	53	35
QLD (11)	–	–	–	22	–	–	44	–	44
SA (3)	–	–	67	33	33	33	33	67	67
VIC (7)	100	–	71	57	–	57	100	86	71
WA (9)	–	67	56	–	11	33	22	44	44
**total**^a^	**92**	**75**	**67**	**42**	**30**	**57**	**50**	**54**	**45**
Total^b^ (97)	92	74	66	51	36	53	55	63	45

^a^ Reports only included for jurisdictions that include the principle in planning documents

^b^ All reports included irrespective of whether the principle is stated in planning documents

## Discussion

In this paper we reviewed Australian Commonwealth and State and Territory government health planning policy to find out what principles governments state as being important to the work they do in Aboriginal and Torres Strait Islander contexts. We then assessed whether these principles are reflected in evaluation commissioning and evaluation practice. We found that despite a strong rhetoric that emphasised the importance of partnership and holism and to a lesser degree cultural competency and capacity building, these principles were not widely reflected in evaluation practice for the period 2007–2017.

For Commonwealth and State and Territory governments, establishing a solid evidence base that centres Aboriginal and Torres Strait Islander perspectives and understandings across the policy cycle (planning, implementation, evaluation) were stated as goals of the work they do. We found that principles such as evidence-based, accountability and equity underpinned most of the evaluations. Despite all seven health plans including the principle of partnership approaches, there was imperfect evidence of this in practice. The mechanisms to centre Aboriginal and Torres Strait Islander perspectives and understandings to fulfil the principle of partnership were less frequently embedded in evaluation practice. We note that although evaluations often engaged Aboriginal people or communities (i.e., consulted with key people or had an Aboriginal reference group), engagement as a mechanism for including Aboriginal perspectives is not always as far reaching as partnerships where the ACCS is at least theoretically provided ownership of decision making. However, in saying this we remain mindful that the process of colonisation impacts the power dynamic between Aboriginal partner/s and any dominant institution, where economic, resourcing and political power is largely held by the later [28]. In health planning, the meaning of engagement encompassed concepts from consulting through to community control, but rarely did we see Aboriginal community controlled organisations leading or enacting the principle of self-determination over evaluation. Although not represented in government health planning documents, many have asserted the importance of embedding the principle of self-determination in evaluation so that Aboriginal perspectives can be centred [29, 30]. Examples of evaluations that centre self-determination principles in utilising the skills, strengths and knowledge of the community-controlled sector are rare in contemporary practice [29–33]. Indeed, NACCHO (2019) raises concerns that evaluations are largely dominated by a private sector that is spatially and culturally removed from Aboriginal people and organisations, and question how such practice can centre Aboriginal perspectives, knowledge and experiences [29].

Our finding that the principles of equity, accountability and evidence-based most frequently underpin evaluations is best understood in terms of wider Commonwealth policy. The 2007–2017 period was marked by the Closing the Gap policy agenda and its overarching objective to build a robust evidence base, addressing disparities and achieving health equity for Aboriginal and Torres Strait Islander people. In recent years there have been increasing criticisms that Closing the Gap policy lacks the perspective of Aboriginal people through the decade-long exclusion of key Aboriginal bodies such as NACCHO from leading policy formation [34]. Policy without the strong voice of the ACCS has meant that diverse aspirations of all Aboriginal people have been excluded in favour of policy whose objectives are only equitable for Aboriginal people who aspire to live under socio-cultural standards set by the dominant culture [35, 36]. The full breadth of aspirations of Aboriginal people can only be responded to when these voices formulate policy. The recent 2019 Closing the Gap Partnership Agreements signed by the Commonwealth Government, State and Territory Governments and the Coalition of Aboriginal and Torres Strait Islander Peak Bodies has been a recent development to increase ACCS participation and involvement in Closing the Gap policy [37]. While peak bodies have long advocated for Aboriginal leadership in defining policy, health bodies such as NACCHO have also called for Aboriginal definitions of successful programs and policy to be given weight, alongside traditional evidence-based approaches [29].

In addition to lacking Aboriginal perspectives, evaluations infrequently centred Aboriginal understandings of health, despite health planning documents stating that a holistic understanding of health should underpin the work being done. Rather than drawing on NACCHOs definition of health adapted from the National Aboriginal Health Strategy (1989) (see Table 2) [27], evaluations instead focused on individual and biological outcomes (i.e., change in disease rates, behaviours or knowledge). We note that even when a holistic concept of health was present it was not well interpreted. It was not uncommon for evaluators to alter the concept of holistic health and present dominant social determinants, or individualised mental health measures. These findings echo a review by Lutschini (2005) that reported that despite the holistic concept of health being centrally placed in policy and strategies, policy makers often lacked a coherent articulation of the concept, were often uncritical and unreflective in their use and interpretation of it and often altered the concept and constitutional element without justification [38]. In saying this, we recognise that in the absence of widely accepted and valid quantitative measures for holistic health and wellbeing, evaluating for the holistic concept of health is difficult [39]. But we also know of the increasing scholarship in recent years to capture concepts of holistic health using narrative methods and Indigenous methodologies [40].

Across the Australian health planning documents there were not always consistent articulations of what was meant by the principles of shared responsibility, cultural competence, engagement, partnership, capacity building, equity, a holistic concept of health, accountability, or evidence-based. There were also varying degrees to which these articulations engaged with Aboriginal understandings and preferred articulations of these concepts.

A limitation of our review is that the principles we are evaluating are those that governments state as important to the work they do. Although such principles would have been developed through consultation with the ACCS, they may not capture those principles that are most important to Aboriginal people and the ACCS. We are cognisant that in neo-colonial contexts, health policy, including evaluation, cannot be separated from the control and regulation of Aboriginal bodies [6]. There is, without a doubt, an imbalanced power dynamic in current policy that sees dominant cultures policing Aboriginal populations and controlling the health agenda. As such, improving evaluation practice requires more than just governments doing what they say they will. As recognised by the Productivity Commission, there is a real need for transformative reform. We highlight that some of the principles that the ACCS have highlighted are important to evaluation and policy more broadly. These include: self-determination, community control, rights based approached, Aboriginal-led, investment in Aboriginal capacity, strengthen-based, do no harm, ethical, effective, transparent, cultural continuity, recognition of systems inequalities, recognition of past colonising and culturally safe evaluations, community benefit, transformative/decolonising orientations, social justice, Aboriginal cultural and intellectual property rights, empowering, and recognising diversity. It is obvious that the ACCS want Aboriginal perspectives, experiences and understandings to be central to evaluation policy pertaining to Aboriginal and Torres Strait Islander people [29–33, 41]. In addition, the reference group for this project that included members of the ACCS emphasised the importance of capitalising on Aboriginal strengths and data governance and sovereignty [13].

We also recognise that a limitation of our evaluation is that we only provide a count of the principles that have been reported within publicly available evaluations put out for advertised tenders by Australian governments. It is not clear what level of activity we have failed to capture. Our method of identifying public tenders, has also meant that we do not capture the full breadth of evaluations tendered by Aboriginal community controlled and other organisations. We realise that we are not capturing how such principles relate to internal evaluations, including those done by Aboriginal organisations as part of continuous quality improvement, monitoring and safety activity. In addition, the focus on external independent evaluations means that the evaluators are less likely to be subject to disincentives to reporting poor or adverse outcomes than might be the case in other forms of evaluation.

For evaluations to centre Aboriginal perspectives and understandings, there needs to be greater involvement of Aboriginal and Torres Strait communities and organisations in the planning and implementation of programs, policies and services, not just evaluation [2]. This recognises that many of the terms for evaluation including the outcome measures are decided early in the process of planning a policy, program or service [35]. As rightly identified by Altman (2019), for any principle to be meaningfully embedded in an evaluation there needs to be recognition of it across the policy cycle and importantly at the planning stage, where the parameters of the evaluation are largely set [35]. For example, principles such as self-determination, social justice or anti-racism cannot be fully realised in evaluation if measures of programmatic success are pre-defined by government. To centre Aboriginal perspectives there needs to be mechanisms for Aboriginal and Torres Strait Islander leadership and ownership at all phases of the program planning and evaluation cycle. With recent development of the ‘Ngaa-bi-nya Aboriginal and Torres Strait Islander framework’ and the ‘Lowitja Evaluation framework to improve Aboriginal and Torres Strait Islander health’ there are now frameworks that talk to the ethical responsibilities of doing evaluation in Aboriginal contexts [11, 13]. Use of ethical frameworks that delineate the responsibilities of all parties in evaluation (including commissioners, evaluators, implementors, Aboriginal community, Aboriginal participants) have potential to improve evaluation practice.

The findings we report here also highlight problems of transparency in current tendering and evaluation practice. We found that despite tenders being publicly listed none of the tendering organisations had mechanism for tracking once it was let. We were only able to access 5% of tenders. It is a concern that there is no publicly available repository for tenders as this would provide a means to conduct quality assurance practices to improve practice. We are also cautious that our findings only relate to 5% of all tenders; as such we do not know the true extent to which tendering practice is reflective of health planning. It should also be recognised that the tendering process is one way to change practice. One straightforward mechanism to include Aboriginal perspectives in current practice is for all tenders to explicitly state that a partnership and engagement approach is a requirement for prospective evaluators. This would also be beneficial for capacity building and cultural competency criteria to be included if commissioners are to align their practice with their health planning. Government principles for working with Aboriginal and Torres Strait Islander people should underpin evaluation tendering selection and reporting.

In Aboriginal health, it is widely accepted that evaluation reports remain ‘on the shelf’ and do not inform the next iteration in the health policy cycle. However, we found here that many reports did not even make it ‘to the shelf’ as we found only 1 in 4 reports was publicly accessible [13]. This raises important questions regarding to what extent evidence from evaluation contributes to the next phase of planning in the policy cycle, when it is not easily available, especially to the ACCS. Given that governments place such strong emphasis on evidence-based policy and programs in Aboriginal and Torres Strait Islander health contexts, the evidence must be made available in order to close the policy-evaluation cycle. We propose that transparency and accountability across evaluation can be improved by ensuring public access to tender documents, evaluation reports and documentation of responses to evaluations.

## Conclusions

Our findings quantifiably reflect widespread criticism that current evaluation practice lacks the important perspectives of Aboriginal people and the ACCS, despite UN recognition of the importance of Indigenous participation in their own health. Even with Australian governments acknowledging the importance of Aboriginal perspectives and stating that engagement and partnership will underpin the work they do, this is imperfectly enacted in evaluation practice. Our findings also highlight the need for greater transparency in evaluation practice and accessibility of evaluation findings.

## Data Availability

All data generated or analysed during this study are included in this published article. All analysed materials are publicly available.

## Abbreviations

ACCS: Aboriginal community-controlled sector
NACCHO: National Aboriginal Community Controlled Health Organisation
NHMRC: National Health and Medical Research Council
NSW: New South Wales
NT: Northern Territory
PRG: Project reference group
QLD: Queensland
SA: South Australia
VIC: Victoria
WA: Western Australia
